# Prevalence of overweight, obesity and thinness in 9–10 year old children in Mauritius

**DOI:** 10.1186/1744-8603-8-28

**Published:** 2012-07-23

**Authors:** Rishi Caleyachetty, Alicja R Rudnicka, Justin B Echouffo-Tcheugui, Karen R Siegel, Nigel Richards, Peter H Whincup

**Affiliations:** 1 King’s College London School of Medicine, University of London, London, UK; 2 St Edmund's College, University of Cambridge, Cambridge, CB3 0BN, UK; 3 Division of Population Health Sciences & Education, St George’s, University of London, London, UK; 4 Department of Global Heath, Rollins School of Public Health, Emory University, Atlanta, USA; 5 Centre for Applied Social Science Research, University of Mauritius, Reduit, Mauritius

**Keywords:** Body mass index, Children, Mauritius, Obesity, Thinness

## Abstract

**Objective:**

To document the prevalence of overweight, obesity and thinness in 9–10 year old children in Mauritius.

**Methods:**

412 boys and 429 girls aged 9–10 years from 23 primary schools were selected using stratified cluster random sampling. All data was cross-sectional and collected via anthropometry and self-administered questionnaire. Outcome measures were BMI (kg/m^2^), prevalence of overweight, obesity (International Obesity Task Force definitions) and thinness (low BMI for age). Linear and logistic regression analyses, accounting for clustering at the school level, were used to assess associations between gender, ethnicity, school location, and school's academic performance (average) to each outcome measure.

**Results:**

The distribution of BMI was marginally skewed with a more pronounced positive tail in the girls. Median BMI was 15.6 kg/m^2^ in boys and 15.4 kg/m^2^ in girls, respectively. In boys, prevalence of overweight was 15.8% (95% CI: 12.6, 19.6), prevalence of obesity 4.9% (95% CI: 3.2, 7.4) and prevalence of thinness 12.4% (95% CI: 9.5, 15.9). Among girls, 18.9% (95% CI: 15.5, 22.9) were overweight, 5.1% (95% CI: 3.4, 7.7) were obese and 13.1% (95% CI: 10.2, 16.6) were thin. Urban children had a slightly higher mean BMI than rural children (0.5 kg/m^2^, 95% CI: 0.01, 1.00) and were nearly twice as likely to be obese (6.7% vs. 4.0%; adjusted odds ratio 1.6; 95% CI: 0.9, 3.5). Creole children were less likely to be classified as thin compared to Indian children (adjusted odds ratio 0.3, 95% CI: 0.2, 0.6).

**Conclusion:**

Mauritius is currently in the midst of nutritional transition with both a high prevalence of overweight and thinness in children aged 9–10 years. The coexistence of children representing opposite sides of the energy balance equation presents a unique challenge for policy and interventions. Further exploration is needed to understand the specific causes of the double burden of malnutrition and to make appropriate policy recommendations.

## Background

The nutrition transition describes the process in which major shifts in patterns of diet and physical activity occur over time although the stage and speed of transition varies by country. A key feature of the nutrition transition is the coexistence of dual expressions of under-and overnutrition referred to as the “double burden of malnutrition” [1].

It is commonly asserted that low- to middle-income countries (LMICs) are characterized by the double burden of malnutrition [2-4], although Corsi et al.. recently reported that the hypothesized “double burden” of malnutrition has not occurred in a substantial manner in either among adult women of reproductive age or among adult males in a majority of the LMICs.

Globally overnutrition is increasing in children while undernutrition decreases in many countries [5]. However among children across Sub-Saharan African countries, underutrition remains the greatest problem rather than overnutrition [6]. Few studies have reported the presence of the double burden of malnutrition *within* the same children [7-10]. This phenomenon has not yet been documented in Mauritius, a middle-income country located off the south-east coast of Africa, with a very diverse population composed of south Asian Indian (68%), Creole (predominantly of African and Malagasy ancestry with some European admixture) (27%), Chinese (3%) and Caucasians (3%). The consequences of overweight and obesity are particularly important in children of Indian and African origin as healthy children from these ethnic groups have been reported to be more insulin resistant than white children [11,12]. Thinness in older children has been associated with delayed pubertal maturation, reduced muscular strength and work capacity [6]. Prevalence of overweight and obesity in adult Mauritians increased during the 1987–1992 period from 26.1% to 35.7% in men and from 37.9% to 47.7% in women [13]. The country also has one of the highest prevalence of type 2 diabetes globally, with over 20% of adults over 30 years of age with type 2 diabetes and 20% with pre-diabetes [14].

Describing the extent of the double burden of overnutrition and undernutrition in children has clear implications for informing policy and design of interventions in Mauritius. This paper reports the prevalence of overweight, obesity and thinness in Mauritian children age 9–10 years.

## Methods

We performed a cross-sectional study of urban and rural primary children aged 9–10 years from a representative sample of primary schools in Mauritius. The study was completed during August and September 2006. In order to detect an expected obesity prevalence of 5%, with a standard error of about 1%, approximately 900 children were recruited to the study. A stratified self-weighted cluster sampling design was used to recruit children. Schools were stratified according to the four educational zones in Mauritius, because the education system is partially decentralized with a regional office in each of the four zones. From each stratum, six schools were randomly selected. If a school declined to participate in the study it was replaced by the next school on the list. Within the schools, a fixed proportion of children aged 9–10 years were systematically randomly selected from class registers.

The study was approved by the Mauritian Ministry of Education and Human Resources. Children gave verbal consent and their parent/guardian gave written informed consent.

Data collection included a short self-administered socio-demographic questionnaire in English and anthropometric measurements. Height was measured to the nearest 0.1 cm with a Seca Leicester Portable Height Measure. Weight was recorded to the nearest 0.1 kg using Tanita digital scales (Tanita Corporation, Tokyo, Japan). Both height and weight measurements were performed while children were lightly dressed and without shoes.

Body mass index (BMI) was calculated by weight (kg) divided by height squared (m^2^). Age–appropriate International Obesity Task Force cut-off values of BMI were used to define overweight and obesity; these are equivalent to the adults cut-off points of less than or equal to 25 kg/m^2^ and 30 kg/m^2^, respectively [15]. To classify children as underweight or “thin”, we used a recent classification system by Cole et al.., which uses age specific cut-offs for BMI in childhood to pass through a given BMI at age 18 years [16]. For our analyses, we used Cole’s definition of grade 2 thinness, which is defined as less than or equal to 17 kg/m² at age 18 years. We chose grade 2 because the BMI cut-offs in childhood are close to z-score −2 which can be assumed to correspond reasonably well with the z-score of −2 of weight for height in children, which is the World Health Organization (WHO) criteria for malnutrition and wasting in children [16]. Additionally, the BMI cut-off of 17 kg/m² in adulthood is close to the WHO definition of grade 2 thinness in adults (BMI < 17 kg/m²), allowing for comparisons of our data with other data from other countries [16]. Urban area was defined using the Mauritian government criteria, as towns larger than 50,000 inhabitants [17]. We classified schools into three approximately equal sized groups on the basis of the proportion of children passing the Certificate of Primary Education (CPE)- a measure of academic achievement in that school in the previous year. In Mauritius there is an excessive emphasis on passing the CPE and getting to a good secondary school, often leading to a situation where students are required to seek private tuition [18]. Thus, school's academic performance was used a proxy for a school environment that may excessively displace out-of-school physical activity.

Linear regression analysis was used to examine the association of BMI with gender, ethnic origin of the child (Creole vs. Indian), whether the schools resided in an urban or rural environment and level of school achievement. To account for a marginally asymmetric distribution of BMI, we ran the linear regression after a log transformation, but this did not substantially improve the distribution of residuals from the regression model. Thus, we reported the non-transformed linear model. Logistic regression analysis was used to examine the relation of the same correlates to the prevalence of obesity, overweight and thinness. All models took into account the clustering of individuals within schools by using robust standard error estimation. Estimates are presented from models that mutually adjusted for all correlates considered along with 95% confidence intervals (CI). All analyses were performed using Stata Version 9 (Stata Corp, College Station, Texas).

## Results

Of the 24 primary schools approached, 23 (96%) participated in the study and a total of 841 children (412 boys and 429 girls, 29–41 per school, estimated response rate 70%) participated. The median age of participants was 10.0 years (standard deviation ± 0.4). Most children were either Indian (79%) or Creole (17%). Non-responses was due to children being absent on the days of survey.

**Figure 1:**
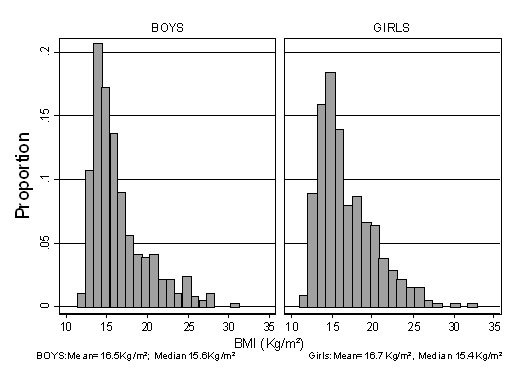
Distribution of BMI in boys and girls separately.

Overall 17.4% of participating children were overweight, 5.0% were obese, and 12.7% per cent were thin. There was no evidence of any significant difference in the prevalence of overweight, obesity or thinness between girls and boys (Table 1). However, on average girls were significantly heavier and taller than boys (p < 0.05). The distribution of BMI is marginally skewed (Figure 1) with a more pronounced positive tail in the girls. Median BMI was also slightly but not statistically significantly higher among girls than boys (p = 0.38). Mean BMI did not differ appreciably between African and Indian children. Prevalence of overweight and obesity were both slightly but not significantly lower among African than Indian children (Tables 2 and 3). African children were markedly less likely to be classified as underweight compared to Indian children (5.6% vs. 14.6%).

**Table 1:** Anthropometric characteristics and prevalence of overweight, obesity and thinness in 9–10 year old children by sex

**Outcome**	**Boys (N = 412)**	**Girls (N = 429)**	**p-value‡**
	**Median (95% CI†)**	**Median (95% CI†)**	
Weight (kg)	30.0 (29.9, 30.8)	31.3 (30.5, 32.1)	0.05
Height (cm)	138.9 (138.1, 139.8)	140.6 (139.9, 141.6)	0.003
BMI (kg/m^2^)	15.4 (15.2, 15.8)	15.8 (15.4,16.1)	0.38
	Prevalence estimates (%)		
	% prevalence (95% CI)	% prevalence (95% CI)	
Overweight	15.8 (12.6, 19.6)	18.9 (15.5, 22.9)	0.23
Obesity	4.9 (3.2, 7.4)	5.1 (3.4, 7.7)	0.86
Thinness	12.4 (9.5, 15.9)	13.1 (10.2, 16.6)	0.77

Note. CI = confidence interval.

†95% confidence intervals for medians are binomial exact.

‡p-value from *t*-test for comparison of means and χ² test for comparison of prevalence estimates.

**Table 2:** Body mass index and prevalence of overweight, obesity and thinness in 9–10 year old children

**Correlate**	**N**	**Mean BMI (Standard error)**	**Prevalence**		
**Ethnic group**		**kg/m**^**2**^	**Overweight**	**Obesity**	**Thinness**
Indian	665	16.6 (0.13)	18.2%	5.6%	14.6%
Creole	143	16.8 (0.29)	16.8%	3.5%	5.6%
Other†	33	16.0 (0.60)	3.0%	-	6.1%
p-value		0.48	0.03	0.29	0.003
School setting					
Urban	298	16.9 (0.15)	18.1%	6.7%	11.1%
Rural	543	16.4 (0.20)	16.9%	4.0%	13.6%
p-value		0.04	0.67	0.10	0.28
School achievement					
Low	311	16.3 (0.19)	16.1%	4.5%	13.2%
Average	263	16.5 (0.21)	17.1%	3.8%	12.9%
High	267	16.9 (0.21)	19.1%	6.7%	12.0%
p-value for trend		0.05	0.34	0.24	0.67

p-values are from *t*-test for comparison of means and χ² test for comparison of prevalence estimates.

^†^This included children that were of Chinese (N = 3) and unknown ethnic group (N = 30).

**Table 3:** Association of Body mass index (BMI) and prevalence of overweight, obesity and thinness with correlates

**Correlate**	**Difference in BMI**	**Odds ratios (95% CI)**		
	**kg/m**^**2**^**(95%CI)**	**Overweight**	**Obesity**	**Thinness**
Girls vs. Boys	0.2 (−0.3, 0.7)	1.23 (0.83, 1.84)	1.05 (0.56, 2.08)	1.09(0.73, 1.63)
Creole vs. Indian	0.3 (−0.3, 1.0)	0.93 (0.57, 1.53)	0.64 (0.21, 1.97)	0.33(0.19, 0.57)
Urban vs. Rural	0.5 (0.01, 1.0)	1.12 (0.83, 1.51)	1.62 (0.87, 2.99)	0.84(0.52, 1.34)

Note. CI = confidence interval.

All estimates from regression models are mutually adjusted for the factors listed in the table taking account of clustering of individuals within school.

Children attending urban schools had a significantly higher mean BMI than children attending rural setting (16.9 kg/m^2^ vs. 16.4 kg/m^2^) (Table 2). Children attending urban schools were more likely to be overweight (adjusted odds ratio = 1.12; 95% CI: 0.83, 1.51) or obese (adjusted odds ratio = 1.62; 95% CI: 0.87, 2.99) than those attending rural schools, although these differences were not statistically significant (Table 3). There was a positive association between BMI and a schools’ academic performance, with a trend of increasing levels of BMI in schools where a higher proportion of children passed the CPE in the previous year (p-value for trend = 0.05). Differences in the prevalence of overweight, obesity and thinness were not statistically significant either in unadjusted or adjusted analyses (p-value for trend >0.4 for all three outcomes).

## Discussion

This study shows that Mauritius is currently in the midst of nutritional transition, with both a high prevalence of overweight and thinness in 9–10 year old children. Nearly one-fifth of the children in this sample were overweight, 5.0% were obese, and 12.7% of children were thin. Prevalence of overweight and/or obesity did not vary by gender or by ethnicity, but prevalence of thinness was higher among Indian children. Prevalence of overweight and obesity was higher among urban children and in children attending schools with higher academic achievement scores.

The simultaneous prevalence of overnutrition (i.e. overweight or obesity) and undernutrition (i.e. stunting) within the same group of children has been previously reported in LMICs [7-10] and regions [6] undergoing the nutritional transition. For example in the African region in children aged 6–12 years, the mean prevalence of overweight and obesity was 7% and thinness 36% [6]. The much lower prevalence of overweight and higher prevalence thinness compared to Mauritius is probably related to the country’s economic performance (and hence stage of nutrition transition). Between 1977 and 2009, real GDP in Mauritius grew on average by 5.1% annually, compared with 3.2% for Sub-Saharan Africa [19]. In 2010, the Mauritian Government reported prevalence estimates in the 5–11 year age group using national survey data collected in 2004 [20]. The prevalence of overweight was 7.9% and 8.5% for overweight and obesity, respectively, among boys; corresponding values among girls were and 7.5% and 7.8%. However, direct comparison of these estimates with ours is difficult, given the difference in age groups studied, and the lack of readily available details on the design of the national survey, and cut-offs used in the classification of overweight and obesity.

In our study, schools with higher academic achievement scores had higher mean BMIs. This could be because those schools with high academic achievement had more children attending private tuition which may displace out-door physical activities and lead to higher BMI. However, high academic performance could also be a proxy for high socioeconomic status where only those families with greater household incomes could afford private tuition. In Mauritius children aged 9–10 years from wealthier families tend to take more tuition than students from poorer families [18].

Our findings suggested that the coexistence of overweight and thinness was particularly prominent among Indian children in Mauritius. This may be because particular socio-economic groups within the Indian population experience the nutrition transition at varying rates (more so than the Creole population) as some may have insufficient resources to meet their children’s calorie requirements, while others have more than enough resources to meet these requirements [21]. In a large population-based study covering 26 Indian states, Subramanian et al. reported that the double burden of malnutrition in India was more likely to occur in high-inequality states [22].

From the 1970’s, Mauritius has undergone many of the demographic and epidemiological changes associated with the nutrition transition [1,23] including a decline in fertility (which has led to an ageing population profile) and an increase in risk factors for non-communicable disease in a context of rapid urbanization and modernization. The latter is a consequence of sustained economic growth, market integration, and foreign direct investment [19]. These macrolevel drivers also produced changes in diet and physical activity patterns. Diets among educated and wealthier adults in Mauritius follow a western dietary pattern characterized by higher consumption of cakes/pastries, meat and fast foods [24]. The pattern is similar in children, with surveys of school children reporting that most of the popular snacks were high in carbohydrate, fat, or salt [25,26]. Physical activity levels are low for both men and women aged 25–74 years. Data from the 2009 National Non-Communicable Survey reported that only 10.9% of women and 23.2% of men undertook moderate or vi-gorous physical activity each day [14]. Similarly, low levels of physical activity are present in children. The Global School Health Survey in 2007 showed that only 13% of students aged 13 to 15 years were engaged in regular physical activity of at least 60 minutes per day [27].

To our knowledge, this is the first study to assess the prevalence of overweight, obesity and thinness in children, using objectively measured height and weight data from a population-based sample in Mauritius. In addition to a fairly robust design, we used an appropriate and commonly accepted definition of childhood overweight and obesity (International Obesity Task Force) [15] and indicator of undernutrition in older children (thinness- recommended by the WHO) [6].

There are limitations to this study. First, the relatively limited size of the study means that although overall estimates of BMI, overweight, obesity and thinness prevalence are reasonably precise, the study lacks statistical power for subgroup comparisons. However, a much larger study would have required more funds. Second, due to the cross-sectional nature of the study, we could not reliably assess the causal processes of the double burden of malnutrition. Third, if missing participants were underweight, overweight, or obese children prevalence estimates from the sample would underestimate the true population values. Fourth, due to the expected high frequency of non-valid responses [28], children were not asked direct indicators of socioeconomic position and thus we are unable to comment on the socioeconomic patterning of the double burden of malnutrition in Mauritius. Lastly, the finding of little difference between urban and rural areas in all of the outcomes should be viewed with caution given that they may only be little differences between ‘urban’ and ‘rural’ areas in Mauritius now [17]. For e.g. in the 2000 census, approximately a quarter of the population lived in settlements with between 5,000 and 20,000 inhabitants. These settlements included various district capitals that were not classified as urban areas. Mauritius’s population would have been more than two-thirds urban in 2000, if they had been classified as urban centres [17].

## Conclusion

Our study shows a high prevalence of overweight and thinness in 9–10 year old children in Mauritius. The coexistence of children representing opposite sides of the energy balance equation presents a unique difficulty for public health policy and interventions in Mauritius. Further exploration is needed to understand the specific causes of the dual burden condition and to make appropriate policy recommendations.

## Competing interests

The author(s) declare that they have no competing interests.

## Authors’ contributions

RC, ARR, and PHW were responsible for the conception and design of the study. RC and NR was involved in the collection and compilation of data. RC and ARR analyzed the data; RC wrote the first draft of the manuscript, and all authors contributed to its redrafting and have approved the final version. All authors read and approved the final manuscript.
